# TCDD and cancer: A critical review of epidemiologic studies

**DOI:** 10.3109/10408444.2011.560141

**Published:** 2011-07-01

**Authors:** Paolo Boffetta, Kenneth A Mundt, Hans-Olov Adami, Philip Cole, Jack S Mandel

**Affiliations:** 1 International Prevention Research Institute, Lyon, France; 2 ENVIRON International Corporation, Amherst, Massachusetts, USA; 3 Department of Epidemiology, Harvard School of Public Health, Boston, Massachusetts, USA; 4 Department of Epidemiology, School of Public Health, The University of Alabama at Birmingham, Birmingham, Alabama, USA; 5 Exponent Inc., Menlo Park, California, USA

**Keywords:** Cancer, dioxin, epidemiology, TCDD

## Abstract

The authors reviewed the epidemiologic studies on exposure to 2,3,7,8-tetrachlorodibenzo-*p*-dioxin (TCDD) and cancer risk, published since the last full-scale review made by the International Agency for Research on Cancer Monographs program in 1997. The update of a cohort of US herbicide producers generated negative results overall; the internal analysis provided evidence of an increased “all-cancer” risk in the highest exposure category, with a statistically significant exposure-response association in some of the many analyses performed.The update of a similar Dutch cohort did not confirm the previously observed association with TCDD exposure. The updated surveillance of the Seveso population provided evidence of increased all-cancer mortality 15-20 years after exposure among those living in the most contaminated area but might also reflect random variation, as overall excesses in the most recent follow-up were not observed. Corresponding data on cancer incidence offer little support to the mortality results. Updated results from cohort studies of Vietnam veterans potentially exposed to TCDD did not consistently suggest an increased risk of cancer. Results of additional, smaller studies of other occupational groups potentially exposed to TCDD, and of community-based case-control studies, did not provide consistent evidence of an increased cancer risk. In conclusion, recent epidemiological evidence falls far short of conclusively demonstrating a causal link between TCDD exposure and cancer risk in humans. The emphasis on results for overall cancer risk—rather than risk for specific neoplasms—is notjustified on epidemiologic grounds and is nota reason for ignoring the weaknesses of the available evidence.

## Contents

Abstract…………622
Introduction…………623
IARC reviews of carcinogenicity of TCDD in humans…………623
Aims…………623
Methods…………624
Selection of studies…………624
Results subsequent to the IARC 1997 review…………624
Outcomes…………624
Review of epidemiologic studies…………625
Manufacture of herbicides potentially contaminated with TCDD…………625
Update of the multicenter US cohort…………625
Update of the Midland cohort…………625
Update of the Dutch cohort…………626
Update of the New Zealand cohort…………626
Herbicide manufacturers—Conclusions…………627
Application of herbicides potentially contaminated with TCDD…………627
Seveso industrial accident…………627
Vietnam veterans, with emphasis on risk of prostate cancer…………628
Community-based case-control studies of NHL and STS…………630
TCDD exposure and risk of multiple myeloma…………630
TCDD exposure and risk of breast cancer…………630
Studies of chloracne patients…………631
Discussion…………631
Acknowledgments…………633
Declaration of interest…………633
References…………634

## Introduction

2,3,7,8-Tetrachlorodibenzo-*p*-dioxin (TCDD) is carcinogenic in experimental animals, but has not been conclusively proven to cause cancer in humans. Indeed, evidence for an effect in humans has remained controversial. This is in part because exposure to TCDD is widespread, but almost invariably occurs at very low doses as a contaminant, and is typically part of complex mixtures of chlorinated compounds, several of which are also suspected to be carcinogenic. Such exposure circumstances represent a challenge to epidemiology, because of methodological issues such as limited power to detect small risks, exposure misclassification, and residual confounding.

As with other important known or suspected environmental carcinogens, emphasis on the carcinogenic effect of TCDD rests mainly on exposure-response modeling and possible health (and in particular carcinogenic) effects at low levels of exposure. Such effects are generally not observable with epidemiologic methods. Instead, they are often estimated based on low-dose extrapolation models. However, in the absence of empirical data, such extrapolations depend heavily on assumptions regarding the exposure-response relationship at the lowest levels of exposure. Despite their lack of direct relevance to the possible effects at low doses, epidemiologic studies of TCDD and cancer are important because of the need to determine if the agent is carcinogenic to humans exposed to high doses. The question of whether the epidemiologic evidence demonstrates carcinogenicity of TCDD in humans (hazard identification) remains central for risk assessment, and for regulatory decisions.

A number of national and international agencies have reviewed the evidence from epidemiologic studies of TCDD and cancer (e.g., US EPA, 2003; NAS, 2006; WHO, 1998, 2002). In this review, we refer primarily to the reviews conducted by the International Agency for Research on Cancer (IARC) within its Monographs program (IARC, 1977, 1987, 1997; Baan et al., 2009).

### IARC reviews of carcinogenicity of TCDD in humans

The last full-scale IARC Monographs review, completed in 1997, led to a conclusion of limited evidence of carcinogenicity in humans (IARC, 1997). In this review the emphasis was on a small increase in overall cancer risk among humans following high exposure to TCDD, and on exposure-response associations. The overall evaluation leading to classification in IARC Group 1 (established human carcinogen) was based on mechanistic considerations on the role of the aryl hydrocarbon (Ah) receptor in TCDD-related carcinogenesis in both humans and animals. The same mechanism was used to justify emphasis on the risk of all cancers combined rather than specific cancer sites.

The IARC evaluation was criticized by Cole and colleagues (2003) who stressed the lack of an overall increase in cancer risk in humans exposed to TCDD, the inconsistent selection of highly exposed groups in the IARC evaluation, and the weak evidence that the Ah receptor mediates multiorgan carcinogenicity. Cole and colleagues also briefly reviewed the data published after the IARC 1997 review, stressing that no additional evidence had been reported to alter their conclusions. Conversely, another review, authored by some members of the 1997 IARC panel, argued that the data reported after 1997 supported the IARC conclusions (Steenland et al., 2004). The conclusions by Steenland and colleagues were based on positive exposure-response analyses in the US cohort, the evidence of excesses of some cancers in the Seveso accident cohort, and mechanistic data regarding the role of the Ah receptor in TCDD carcinogenicity.

In 2009 IARC reviewed the carcinogenicity of TCDD as part of a systematic reassessment of all agents classified in Group 1 and classified the evidence of carcinogenicity in humans as sufficient, based on increased risk of all cancers combined (Baan et al., 2009). Details of this latter evaluation, based on a more cursory review of the available data than regular IARC Monographs, have not yet been reported, and a comprehensive review of recent epidemiologic data is not available.

### Aims

We provide a detailed review of the epidemiologic studies on cancer risk among individuals exposed to TCDD. With the exceptions of multiple myeloma and breast cancer, for which recent comprehensive reviews are not available, we do not review in detail results published before 1997 because they are extensively reviewed and readily available in the IARC Monograph, which provided a comprehensive summary of the evidence available at that time (IARC, 1997). We also do not address the adequacy of experimental and mechanistic data to support the hypothesis of a central role of Ah receptor activation in TCDD-related carcinogen-esis in humans and in experimental systems (although we address the use of these data to justify the interpretation of epidemiologic studies), nor do we review low-dose extrapolations and risk assessment models for TCDD.

## Methods

### Selection of studies

The most informative populations for which exposure to TCDD is convincingly documented or highly probable include occupational groups involved in the production or use of herbicides potentially contaminated by TCDD (particularly 2,4,5-trichlorophenoxyacetic acid, or 2,4,5-T), other industrial processes entailing potential exposure to TCDD (e.g., trichlorophenol [TCP] manufacture), as well as populations exposed to potentially contaminated intermediates or herbicides via industrial accidents and war-related circumstances.

We also considered community-based studies in which exposure to TCDD (mainly from occupational sources) has been assessed using different approaches, including self-reports, job-exposure matrices, and expert evaluations. In general, these studies are less informative than those based on occupational or accidental exposures because exposures were lower, of shorter duration, and subject to more misclassification. Nevertheless, these studies are included in the review for the sake of completeness.

We do not review studies of workers exposed to herbicides not contaminated by TCDD (e.g., 2,4-dichloro-phenoxyacetic acid, or 2,4-D), to agents contaminated by polychlorinated dibenzo-para-dioxins (PCDDs) without TCDD (e.g., pentachlorophenol [PCP] [Demers et al., 2006]), and to unspecified combinations of pesticides and herbicides (e.g., studies of farmers or pesticide applicators [e.g., Fleming et al., 1999; Swaen et al., 2004]). Nor do we include studies of populations potentially exposed to TCDD, but without detailed exposure information (e.g., pulp and paper workers [McLean et al., 2006], waste incin-eratorworkers [Leem etal., 2003], fishermen [Mikoczy and Rilander, 2009]), and Vietnam Veterans without information on TCDD exposure (Leavy et al., 2006). Because of the abundance of studies with individual-level exposure and outcome assessment, we do not include ecologic studies in which such information is lacking (e.g., Zambon et al., 2007; Poulstrup et al., 2004; Fukuda et al., 2003; Read et al., 2007; Viel et al., 2008a, 2008b).

We identified the relevant literature from the IARC 1997 Monograph (IARC, 1997) and PubMed searches for subsequent publications. We used keywords such as “dioxin,” “TCDD,” “pesticides,” “cancer,” and “neoplasms” as well as specific cancers. We also checked references in recent reviews. We also included a few studies published after the 2009 IARC review.

### Results subsequent to the IARC 1997 review

We aimed to identify subgroups at highest exposure. One such population comprises exposed individuals who developed chloracne, a dermatologic condition caused by high TCDD exposure. Although it is unclear whether mechanisms leading to chloracne (e.g., genetic susceptibility) are also relevant to carcinogenesis, there is little doubt that chloracne patients experienced heavy exposure. In addition, several studies analyzed duration of exposure to TCDD, time since first exposure, and some quantitative or semiquantitative indices of exposure. These data were reviewed in detail because they help in testing the hypothesis of a causal association between TCDD exposure and cancer risk. The 1997 IARC review identified four groups at highest exposure (see below), and emphasized the importance of the results in these groups (IARC, 1997; Steenland et al., 2004). Wherever possible, we identified new results relevant to the high-exposure groups.

When the same population was studied both before and after the 1997 IARC review, we evaluated the specific contribution of the extended follow-up. Whenever possible, we subtracted the number of observed and expected cancer deaths (or cases) of the early report from the updated report; however, when this was not possible, we showed results of the two reports side by side.

### Outcomes

The evaluation by IARC of the carcinogenicity of TCDD is unique because more emphasis is given to all cancers combined (hereafter referred to as “all cancer”) than to specific cancers. We assume that the results for all cancer do not refer to individual cancer sites. The emphasis on all cancer has been justified by the role of activation of the Ah receptor by TCDD as an unspecific mechanism of cancer promotion in humans as well as experimental systems (Gasiewicz et al., 2008; Schwarz and Appel, 2005). This mechanistic interpretation of epidemiologic results has, however, been criticized (Cole et al., 2003). In the IARC evaluations evidence of a causal association with TCDD exposure was considered strongest for lung cancer, non-Hodgkin lymphoma (NHL), and soft-tissue sarcoma (STS) (Baan et al., 2009). Hence, we systematically reviewed the results for these neoplasms. In addition, we reviewed in detail the studies on prostate cancer among Vietnam veterans potentially exposed to TCDD-contaminated herbicides, as well as studies on breast cancer and multiple myeloma, because these cancers have been specifically discussed in previous reviews as possible evidence of dioxin's human carcinogenicity (Brody et al., 2007; Alexander et al., 2007).

**Figure 1:**
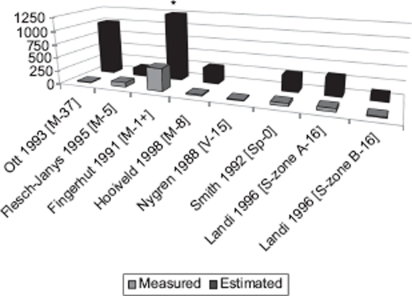
Measured and estimated blood dioxin level (ppt) in selected studies. * 2,000 ppt. In square brackets, type of population (M, pesticide manufacturers; V, Vietnam Veterans; Sp, pesticide sprayers; S, Seveso) and average years between measured and estimated level.

Relatively few measurements are available on levels of exposure of the populations included in the relevant epidemiologic studies. For some of these populations, TCDD was measured in blood samples, usually several years after cessation of exposure. We present selected results in Figure 1, together with the estimated levels at the time of exposure. As a comparison, typical blood levels of TCDD in adults without known sources of exposure fall in the range of 1-10 ppt (IARC, 1997).

## Review of epidemiologic studies

### Manufacture of herbicides potentially contaminated with TCDD

Cancer risk has been extensively studied among workers employed in manufacturing herbicides potentially contaminated with TCDD (mainly 2,4,5-T, TCP, and PCP). These studies differ according to inclusion criteria, follow-up periods, exposure assessment, overlaps, and other characteristics, which complicates comparisons as well as synthesis of evidence across studies. The most comprehensive publication (Kogevinas et al., 1997) combines data from three studies also reported separately: 12 plants from the United States (Fingerhut et al., 1991), 4 plants from Germany (Becher et al., 1996), and 16 plants from Austria, Denmark, Finland, Italy, the Netherlands, New Zealand, Sweden, and the United Kingdom (Saracci et al., 1991). A German cohort exposed through an accident (Ott and Zober, 1996) is the only relevant study not included in the combined international analysis.

Most informative of all these studies—involving the highest and best documented exposure to TCDD—are thel2-plant cohort from the United States, the 4-plant cohort from Germany, the accident cohort from Germany and the cohort from the Netherlands (IARC, 1997; Steenland et al., 2004). Since the 1997 IARC Monograph, updated results have been reported for the US cohort (Steenland et al., 1999, 2001), two subsets of workers in one of the US plants exposed to either TCP/2,4,5-T (Collins et al., 2009a) or PCP (Collins et al., 2009b), the Dutch cohort (Hooiveld et al., 1998; Boers et al., 2010) and the New Zealand cohort ('t Mannetje et al., 2005; McBride et al., 2009).^1^

### Update of the multicenter US cohort

**Figure 2:**
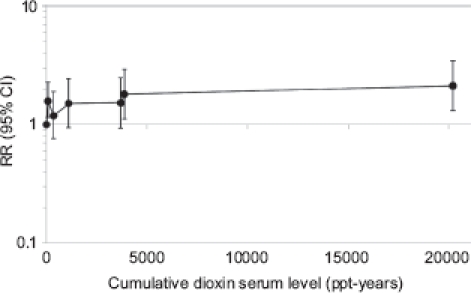
Relative risk of all-cancer mortality in the US cohort, by categories of cumulative dioxin exposure.

As Cole and colleagues (2003) have stated, the update of the US study (Steenland et al., 1999, 2001) provided no evidence of excess cancer mortality in the whole cohort of 5172 workers, beyond what was reported in the first follow-up (Fingerhut et al., 1991) (Table 1). Steenland and colleagues (1999) also analyzed mortality in a subcohort of 608 workers with chloracne (see below), and the results of several analyses based on estimated exposure to TCDD of 3538 workers. In the 1999 article, the results of the main exposure-response analysis (on unlagged cumulative exposure score based on internal comparisons) showed a nonsignificant increase in risk of all-cancer mortality among the workers in the two highest categories of cumulative exposure, whereas there was no gradient in risk across the five categories of lower exposure (Figure 2). Because this cohort experienced a small excess mortality from cancer (all-cancer standardized mortality ratio [SMR] 1.13; 95% confidence interval [CI] 1.02,1.25; essentially reflecting results of the first follow-up) the SMR in the category of highest cumulative exposure is statistically significant (SMR 1.60; 95% CI 1.15, 1.82).

**Table 1:** Comparison of results of the first and the second follow-up of US pesticide manufacturing workers (modified from Cole et al., 2003)

		All cancers		Lung cancer		NHL		STS	
					
Publication	Follow-up	O/E	SMR	O/E	SMR	O/E	SMR	O/E	SMR
Fingerhut et al., 1991	1942–1987	265/229.9	1.15	89/80.1	1.11	10/7.3	1.37	4/1.2	3.38
Steenland et al., 1999	1942–1993	377/*333.6*	1.13	125/*117.9*	1.06	12/*10.9*	1.10	4/*1.7*	2.32
Difference		*112/103.7*	*1.08*	*36/37.8*	*0.95*	*2/3.6*	*0.56*	*0/0.5*	*0*

*Note*. O = observed deaths; E = expected deaths; SMR = standardized mortality ratio; NHL = non-Hodgkin lymphoma; STS = soft-tissue sarcoma.

Figures in italics were derived from raw data reported in the papers.

**Figure 3:**
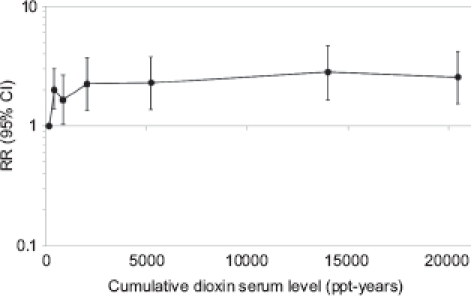
Relative risk of all-cancer mortality in the US cohort, by categories of cumulative dioxin serum level, lagged 15 years.

Authors of the update also analyzed cumulative exposure, log-cumulative exposure, and average exposure and applied various lag times (no lag, 5, 10, 15, and 20 years). A cumulative exposure lagged 15 years showed a statistically significant association (Figure 3 summarizes the results based on cumulative serum TCDD level; similar results were obtained for cumulative exposure score). Mortality from lung cancer was neither increased in the first nor in the updated follow-up (overall SMR 1.06; 95% CI 0.88, 1.26; Table 1); however, an exposure-response association was observed in the analysis by log-cumulative exposure score lagged 15 years (*p* value for trend .03). The excess of NHL and STS mortality in the first follow-up was not confirmed in the extended follow-up (Table 1): no results have been reported on exposure-response analyses for these neoplasms. The 2001 article was based on the same population as the 1999 article, but the results are slightly different and not directly comparable, because the analysis was based on a different exposure index (cumulative TCDD exposure) and different exposure categories.

### Update of the Midland cohort

The updated mortality of two groups of workers in one of the factories of the multicenter US study located in Midland, Michigan, was reported separately (Collins et al., 2009a, 2009b).^2^ The first group of 1615 workers were exposed to TCDD in the manufacture of TCP or 2,4,5-T from 1948 to 1982 (Collins et al., 2009a). No excess in mortality from all cancer was observed. Mortality from lung cancer was reduced (SMR 0.7; 95% 0.5, 0.9). The SMR for NHL was 1.3 (95% CI 0.6, 2.5). Excess mortality was observed for leukemia (SMR 1.9; 95% CI 1.0, 3.2; 13 deaths) and STS (SMR 4.1; 95% CI 1.1,10.5; 4 deaths). The only neoplasm showing an increased mortality according to estimated TCDD exposure was STS. The second group of 773 workers was involved in the manufacture of PCP (Collins et al., 2009b). No excess mortality from all cancer or lung cancer was observed. NHL mortality was increased, based on 8 deaths (SMR 2.4; 95% CI 1.0, 4.7). One death from STS was observed (SMR 2.2; 95% CI 0.0, 12.1). Similar results were obtained when 196 workers with TCP exposure (and included in the TCP subcohort) were excluded.

### Update of the Dutch cohort

The Dutch study comprised workers in two factories included in the multicenter IARC study (Saracci et al., 1991; Kogevinas et al., 1997). Both factories included groups of workers exposed and unexposed to TCDD. In the most recent publication, relative risks (RRs) were calculated by comparing the mortality in the two groups. A comparison of the results with the previous results for factory A (Hooiveld et al., 1998) is possible (Table 2); however, in factory B, only 4 deaths occurred in the first follow-up among exposed workers, and 5 among unexposed workers (Kogevinas et al., 1992), making a comparison with the updated results difficult.

**Table 2:** Comparison of results of the first and the second publication of pesticide manufacturing workers from the Netherlands (factory A)

		*N* workers	All cancers		Lung cancer		NHL	
					
Publication	Follow-up	E/U	E/U	RR	E/U	RR	E/U	RR
Hooiveld et al., 1998	1955–1991	549/482	51/7	4.1	14/1	6.5	3/1	1.7
Boers et al., 2010	1955–2006	539/482	81/31	1.31	20/7	1.15	4/3	0.92

*Note*. E = exposed; U = unexposed; *N* = number of cases; RR = relative risk; NHL = non-Hodgkinlymphoma.

In factory A, the excess risk among exposed workers reported in the early publication (Hooiveld et al., 1998) was not confirmed in the recent update (Boers et al., 2010). As mentioned above, factory A workers were among the groups considered at high TCDD exposure identified in the IARC 1997 review (IARC, 1997; Steenland et al., 2004). In a subset of 140 workers in factory A, employed during an accident resulting in high TCDD exposure, the RR of all cancer was 1.56 (95% CI 0.86, 2.80) compared to the unexposed. No exposure-response gradient was observed in an analysis based on modeled TCDD exposure (Hooiveld et al., 1998). In a recent follow-up of factory B, the RR was 1.54 (95% CI 1.00, 2.37) for all-cancer mortality, and 1.22 (95% CI 0.56, 2.66) for lung cancer (Boers et al., 2010). The reason for the important difference in results between the two reports of mortality among factory A workers Between the two reports the number of observed cancer deaths increased 1.6-fold in the unexposed and more than 4-fold in the exposed (Table 2) is unclear.

### Update of the New Zealand cohort

The follow-up of a cohort of pesticide manufacturers from New Zealand, included in the IARC multicenter study (Saracci et al., 1991; Kogevinas et al., 1992, 1997), has been updated twice since the 1997 IARC Monograph, 't Mannetje et al. (2005) updated the cohort through 2000. The results (Table 3) show a small excess in all-cancer mortality compared to the national rate over time, and increased lung cancer mortality. Duration of employment was not associated with cancer mortality ('t Mannetje et al., 2005). McBride et al. (2009) expanded and further updated this cohort of New Zealand TCP and 2,4,5-T manufacturers. A total of 1599 workers (versus 813 in the previous update) employed between 1969 and 1988 were followed through the end of 2004. Among 1134 exposed workers, the SMR for all-cancer mortality was 1.1 (95% CI 0.9, 1.4). Hie SMR for NHL was 1.6 (95% CI 0.3, 4.7), whereas the lung cancer SMR was 0.8 (95% CI 0.4, 1.5). No significant trends with exposure levels were reported (McBride et al., 2009).

**Table 3:** Comparison of results ofthefirst and the second follow-up of pesticide manufacturing workers from New Zealand

			All cancers		Lung cancer		NHL	
					
Publication	Follow-up	*N* workers	O/E	SMR	O/E	SMR	O/E	SMR
Kogevinas et al., 1992	1969-1987	782	15/11.99	1.25	2/3.56	0.56	0/0.32	0
't Mannetje et al., 2005	1969-2000	813	43/34.6	1.24	12/8.8	1.37	1/1.1	0.87
Difference			*28/22.6*	*1.24*	*10/5.2*	*1.92*	*1/0.8*	*1.25*
McBride et al., 2009	1969-2004	1134	196/196	1.0	61/55.5	1.1	3/1.9	1.6
Difference			*181/184*	*1.0*	*59/51.9*	*1.1*	*3/1.6*	*1.9*

*Note*. O = observed deaths; E = expected deaths; SMR = standardized mortality ratio; NHL = non-Hodgkinlymphoma.

Figures in italics were derived from raw data reported in the papers.

### Herbicide manufacturers—Conclusions

The results of studies of manufacturers of herbicides potentially contaminated with TCDD, reported since the 1997 IARC Monograph review, provide weak additional evidence of an increased all-cancer risk among exposed workers. As already noted (Cole et al., 2003), the update of the US cohort study produced negative results overall; the internal analysis provided evidence of an increased risk in the category at highest exposure, with a statistically significant exposure-response association in some of the many analyses performed. Update of the Dutch cohort did not confirm the association with TCDD exposure previously observed in one factory, and resulted in a small excess risk in the other factory. The other two studies of workers at high TCDD exposure (4-plant German cohort and German accident cohort) have not been updated.

### Application of herbicides potentially contaminated with TCDD

The IARC multicenter study included four cohorts of herbicide sprayers from Australia, Canada, New Zealand, and United Kingdom (Saracci et al., 1991; Kogevinas et al., 1992, 1997). The cohort from New Zealand was independently updated in parallel to the cohort of manufacturers (see above) ('t Mannetje et al., 2005; McBride et al., 2009). No excess mortality from all cancer, lung cancer, or NHL was observed either in the original follow-up of this cohort or in the two updated reports.

In Sweden, 15 foremen, 139 male lumberjacks, and 103 female lumberjacks exposed to 2,4,5-T were followed from 1958 to 1992 (Thörn et al., 2000). The standardized incidence ratio (SIR) for all cancer was 2.74 (95% CI 1.00, 5.96) for foremen, 0.72 (95% CI 0.37,1.25) for male lumberjacks, and 0.82 (95% CI 0.42,1.44) for female lumberjacks. Results for individual cancers were based on small numbers of observed and expected cases; two cases of NHL occurred among lumberjacks versus 0.85 expected.

Overall, the studies of herbicide applicators published after 1997 do not support the hypothesis of an association between indicators of TCDD exposure and cancer risk.

### Seveso industrial accident

In the population exposed to TCDD as a result of the 1976 accident in the TCP production plant in Seveso, Italy, updates of cancer mortality (to 2001) and incidence (to 1996) have been recently reported (Pesatori et al., 2009; Consonni et al., 2008).^3^ This population was divided among those living in zone A (*N* = 723, median serum TCCD level in 1976, 447.0 ppt), zone B (*N* =4821, median serum TCDD 94.0 ppt), and zone R (*N* = 31,643, median TCDD level 48.0 ppt). Neither study reported an excess of all cancer (SMR 0.69 and 1.03) among residents in any zone including zone A, the one with highest soil contamination. However, comparison of results of the mortality report (Consonni et al., 2008) along with the publications available at the time of the 1997 IARC review (Bertazzi et al., 1996) suggests an excess mortality from all cancer, lung cancer, and NHL over time, but not STS (no deaths in zones A and B) (Table 4). This apparent increase reflects a return of observed cases from a slight deficit originally reported to the expected number. Mortality from multiple myeloma was increased among zone A residents (4 deaths at the latest follow-up, SMR 4.34).

**Table 4:** Comparison of results of the last and next-to-last follow-up of residents in Seveso—Cancer mortality

		All cancers		Lung cancer		NHL	
				
Publication	Follow-up	O/E	SMR	O/E	SMR	O/E	SMR
Zone A							
Bertazzi et al., 1996	1976-1991	*16/23.3*	*0.69*	*4/4.5*	*0.89*	*0/0.2*	*0*
Consonni et al., 2008	1976-2001	42/40.8	1.03	11/*8.7*	1.26	3/*0.9*	3.35
Difference		*26/17.4*	*1.49*	*7/4.2*	*1.65*	*3/0.7*	*4.44*
Zone B							
Bertazzi et al., 1996	1976-1991	*152/147.9*	*1.03*	*36/31.7*	*1.14*	*2/2.5*	*0.79*
Consonni et al., 2008	1976-2001	244/*265.2*	0.92	62/55.9	1.11	7/5.7	1.23
Difference		*92/117.3*	*0.78*	*26/24.2*	*1.07*	*5/3.2*	*1.58*
Zone R							
Bertazzi et al., 1996	1976-1991	*1008/1120.0*	*0.90*	*205/224.6*	*0.91*	*18/18.0*	*1.00*
Consonni et al., 2008	1976-2001	*1848/1905.2*	0.97	*383/390.8*	0.98	*40/40.4*	0.99
Difference		*840/785.2*	*1.07*	*178/166.3*	*1.07*	*22/22.4*	*0.98*

*Note*. O = observed deaths; E = expected deaths; SMR = standardized mortality ratio; NHL = non-Hodgkin lymphoma.

Figures in italics were derived from raw data reported in the papers.

Similarly, neither of the two reports on cancer incidence (Pesatori et al., 2009; Bertazzi et al., 1993) demonstrated any excess risk of all cancer (or lung cancer) in any zone in either time period. Between the two time periods there was a small increase in all cancer and lung cancer in zone A residents (Table 5). This apparent increase largely offsets deficits reported in the earlier report (Bertazzi et al., 1993). All-cancer incidence was below expectation at the previous follow-up, but close to expectation at the new follow-up, based on 30 additional observed versus 25.7 expected cases. For lung cancer there were 5 observed and 3.5 expected cases between the two study periods. Results on time since accident (Consonni et al., 2008) are broadly consistent with the results shown in Tables 4 and 5 (because most of the “difference” in Tables 4 and 5 is the result of the follow-up 15 years after the accident), although the numbers are not identical. The excess in all-cancer mortality is apparent only 20+ years after the accident, i.e., after 1996, the year the most recent cancer incidence follow-up ended. However the excess mortality from lung cancer and NHL is apparent also 15–19 years after the accident, without a corresponding increase in the incidence of these two neoplasms, suggesting some inconsistencies between the mortality and the incidence results. For example, misclassification on death certificates may contribute to an excess of mortality not supported by a parallel increase in incidence. Etiologically, the incident cases therefore might provide more valid results.

**Table 5:** Comparison of results of the last and next-to-last follow-up of residents in Seveso—Cancer incidence

		All cancers		Lung cancer		NHL	
				
Publication	Follow-up	O/E	SIR	O/E	SIR	O/E	SIR
Zone A							
Bertazzi et al., 1993	1977-1986	*14/17.0*	*0.82*	*2/2.7*	*0.74*	*0/0.4*	*0*
Pesatori et al., 2009	1977-1996	44/*42.7*	1.03	7/*6.3*	1.12	1/*1.3*	0.80
Difference		*30/25.7*	*1.17*	*5/3.5*	*1.41*	*1/0.9*	*1.18*
Zone B							
Bertazzi et al., 1993	1977-1986	*112/114.1*	*0.98*	*18/17.8*	*1.01*	*4/2.4*	*1.65*
Pesatori et al., 2009	1977-1996	270/*270.0*	1.00	37/*38.5*	0.96	12/7.9	1.51
Difference		*158/155.9*	*1.01*	*19/20.8*	*0.91*	*8/5.5*	*1.47*
Zone R							
Bertazzi et al., 1993	1977-1986	*765/850.0*	*0.90*	*112/130.7*	*0.86*	*22/17.6*	*1.25*
Pesatori et al., 2009	1977-1996	1808/*1883.3*	0.96	280/*269.2*	1.04	49/*54.4*	0.90
Difference		*1043/1033.3*	*1.01*	*168/138.6*	*1.21*	*27/36.9*	*0.73*

*Note*. O = observed cases; E = expected cases; SIR = standardized incidence ratio; NHL = non-Hodgkin lymphoma.

Figures in italics were derived from raw data reported in the paper.

In conclusion, updated surveillance of the Seveso population shows increased all-cancer mortality 15–20 years after exposure among those living in the most contaminated area. However, this might reflect random variation, because no excess was observed in the most recent follow-up. Corresponding data on cancer incidence, limited by a shorter duration of follow-up, offer little support to the mortality results. The number of expected events in the highly contaminated area remains too small to allow conclusions for specific cancer sites.

### Vietnam veterans, with emphasis on risk of prostate cancer

Akhtar and colleagues (2004) investigated cancer incidence and mortality between 1950 and 2000 among 1189 US Air Force veterans who handled Agent Orange, a mixture of herbicides contaminated with TCDD (the Ranch Hand [RH] operation).^4^ Among RH veterans, the SIR for all cancer was 1.08 (95% CI 0.91, 1.26) and the SMR was 0.68 (95% CI 0.50, 0.91).^5^ The SIRs for lung cancer and lymphopoietic neoplasms were not increased. As shown in Table 6, these results agree with early reports of this cohort, including those available at the time of the 1997 IARC review (Ketchum et al., 1996).

**Table 6:** Comparison of results of last and next-to-last follow-up of Ranch Hand veterans—Cancer mortality

			All cancers		Lung cancer		NHL	
					
	N	Follow-up	O/E	SMR	O/E	SMR	O/E	SMR
Ketchum et al., 1996	1261	1966–1993	30/33.3	0.90	12/13.0	0.92	1/0.7	1.43
Akhtar et al., 2004	1061*	1966–2000	45/61.7	0.73	21/24.1**	0.87	6/6.3***	0.95

*Note*. O = observed deaths; E = expected deaths; SMR = standardized mortality ratio; NHL = non-Hodgkin lymphoma.

* Whites only

** respiratory cancer

*** lymphohematopoietic neoplasms.

The new analysis (Akhtar et al., 2004) revealed an excess incidence of prostate cancer (SIR 1.46; 95% CI 1.04, 2.00) and melanoma (SIR 2.33; 95% CI 1.40, 3.65) among White RH veterans. However, the incidence of prostate cancer was also increased in a group of 1776 veterans who did not handle Agent Orange (SIR 1.62; 95%CI 1.23, 2.10) as well as veterans who spent no more than 2 years in Vietnam. Among the 82% of cohort members who had serum TCDD measured between 1987 and 1997, there was no evidence of a TCDD exposure-response relation for all cancer, melanoma, or prostate cancer. Subsequent analyses showed a RR of 1.0 (95% CI 0.8, 1.4) for cancer mortality among RH veterans compared to veterans who were deployed in units in Southeast Asia not using Agent Orange (Ketchum and Michalek, 2005).

A reanalysis was conducted of serum dioxin among the 1482 veterans who did not handle Agent Orange used as comparison group in the main analyses of the RH study. This analysis suggested a relation between increasing serum TCDD level and risk of all cancer and melanoma, but not respiratory or prostate cancer; the risk of prostate cancer, on the other hand, was positively associated with duration of service in Southeast Asia (Pavuk et al., 2005). Analysis of prostate cancer incidence up to 2003 revealed no association with TCDD exposure (Pavuk et al., 2006). Interpretation of cancer risk among RH Veterans is complicated by the lack of clear correspondence of the study populations, as well as the shift from an internal comparison of air force pilots with and without Agent Orange exposure in the early publications to a comparison of these thoroughly surveyed pilots to the general population in the later publications.

Cypel and Kang (2010) updated to 2005 the analysis by Dalager and Kang (1997) of cancer incidence and mortality of a group of 2872 Army Chemical Corps (ACC) Vietnam veterans. ACC units were responsible for handling and spraying herbicides, including Agent Orange, around the perimeters of military base camps. At the time of the 1997 IARC Monograph review only an early report of mortality among 894 ACC veterans was available, with 6 observed cancer deaths versus 6.6 expected (Thomas and Kang, 1990). In the whole group of ACC Vietnam veterans, the SMR for all cancer, based on national mortality rates, was 1.13 (95% CI 0.95, 1.33; 142 deaths), and that of respiratory cancer was 1.35 (95% CI 1.03, 1.73; 60 deaths). The corresponding SMRs were not increased in a comparison group of 2737 non-Vietnam veterans. The only other outcome category with increased mortality in both groups of veterans was a miscellaneous group, mainly comprising malignant neoplasms from undefined organs. Results for NHL were not reported separately, but mortality from lymphohematopoietic cancers was lower than expected (SMR 0.46, 95% CI 0.17, 0.99; 6 deaths). Mortality from nonmalignant respiratory diseases was increased. When a subgroup of 662 Vietnam veterans who reported in 1999-2000 having sprayed herbicides was compared to 811 Vietnam veterans without such exposure, the RR was 1.00 for all cancer (95% CI 0.53, 1.90), and 1.35 (95% CI 0.53, 3.43) for respiratory cancer.

Chamie and colleagues (2008) studied 13,144 Vietnam veterans from California whose Agent Orange exposure status was self-reported. Incidence of prostate cancer was ascertained from 1998 to 2006, between 27 and 44 years following exposure. The total number of identified prostate cancers was 239 among 6214 exposed and 124 among 6930 nonexposed veterans, yielding a Cox proportional hazard ratio of 2.87 (95% CI 2.31, 3.57). The association was even stronger for high Gleason grade and for metastatic disease. These results are of potential concern. Limitations of this study include ambiguities with regard to the reported design and analysis of the study, increased surveillance of exposed veterans, reclassifica-tion of exposure status at prostate cancer diagnosis, self-report of exposure status after diagnosis, inconsistencies in tables and results, as well as unclear standardization of Gleason scores and clinical staging.

A case-control study conducted at the Department of Veterans Affairs Medical Center included 47 prostate cancer cases identified from medical records and 142 hospital controls (Giri et al., 2004). Agent Orange exposure, based on self-reports from medical records, was reported by 11 cases and 17 controls (odds ratio [OR] 2.06; 95% CI 0.81, 5.23). It is unclear whether this information was collected before or after diagnosis.

A prevalence study was conducted among 400 Vietnam veterans referred for prostate biopsy (Zafar and Terris, 2001). Thirty-two veterans who reported Agent Orange exposure were compared to a sample of 96 unexposed veterans. Prostate cancer was detected in 13 exposed and 33 unexposed veterans *(p* value for the difference in proportion was .15). No association was found between Agent Orange exposure and disease differentiation or age at diagnosis.

In conclusion, cohort studies of Vietnam veterans potentially exposed to TCDD as a contaminant of herbicides do not suggest an increased risk of all cancer, lung cancer, or NHL. Recent analyses of RH veterans showed an increased risk of prostate cancer and melanoma, two neoplasms whose incidence is sensitive to diagnostic intensity. Out of three case-control studies of prostate cancer among Vietnam veterans, no association was found in the only study with exposure assessment before diagnosis, which would avoid recall bias. The detection of prostate cancer strongly depends on the intensity of medical surveillance, and in particular testing with prostate-specific antigen (PSA). Intensive PSA screening results in the detection of a large number of early neoplastic lesions of uncertain clinical significance. Differences in prostate cancer incidence between populations can merely reflect differences in screening practices. In only one study (Chamie et al., 2008) was severity of prostate cancer taken into consideration. Studies on prostate cancer mortality, which avoid the potential problem of detection bias, do not indicate an increased risk of prostate cancer among TCDD-exposed workers: in the pooled international analysis of cohorts of herbicide manufacturers and sprayers the SMR for prostate cancer was 1.11 (95% CI 0.81,1.50) forworkers exposed to TCDD and 1.10 (95% CI 0.71, 1.62) for workers not exposed to TCDD (Kogevinas et al., 1997).

### Community-based case-control studies of NHL and STS

A number of community-based case-control studies included in the 1997IARC review (IARC, 1997) addressed the risk of NHL, STS, and other neoplasms. Exposure to TCDD was based on either self-reports or classification of occupational data. Since the 1997 IARC Monograph, various community-based studies of NHL and STS have been reported.

A study of 207 cases of Hodgkin lymphoma, NHL, and chronic lymphocytic leukemia and 180 population controls was conducted in a rice growing area in Italy, in which phenoxyacetic herbicides, including 2,4,5-T, were widely used in the fields (Fontana et al., 1998). The OR for NHL for employment in rice growing was 1.1 (95% CI 0.1, 19) in men and 1.9 (95% CI 0.6, 6.0) in women. No corresponding results were reported for the other two neoplasms included in the study.

In a study of 110 cases with STS and 227 patients with appendicitis as controls from 16 hospitals in Finland, the concentration of 17 PCDD/PCDFs was measured in subcutaneous fat samples (Tuomisto et al., 2004). The risk of STS was lower in subjects with TCDD level (expressed either as World Health Organization Toxic Equivalents [WHO-TEQ] or 2,3,7,8-TCDD) above the lowest quintile, although no exposure-response was observed. The OR in the highest versus lowest quintile was 0.65 (95% CI 0.22, 1.95) for WHO-TEQ and 0.53 (95% CI 0.20, 1.43) for 2,3,7,8-TCDD.

In a study of NHL in four areas of the US including 1321 cases and 1057 population controls, total dioxin, analyzed in plasma samples from a subset of 100 cases and 100 controls, was not associated with NHL risk (OR for 0.01 mol/glipid, 1.002; 95% CI 0.999, 1.005); the corresponding OR for dioxin TEQ was 1.94 (95% CI 0.94, 4.00) (De Roos et al., 2005).

Overall, community-based studies reported after the 1997 IARC review do not support the hypothesis of an association between TCDD exposure and risk of NHL or STS.

### TCDD exposure and risk of multiple myeloma

Incidence of, or mortality from, multiple myeloma among populations exposed to TCDD have been reported in eight studies published after the IARC 1997 Monographs (IARC, 1997). In four of them (Dutch pesticide manufacturers [Boers et al., 2010], New Zealand pesticide sprayers ['t Mannetje et al., 2005], Swedish forestry workers [Thorn et al., 2000], and RH Vietnam Veterans [Akhtar et al., 2004]) no incident cases or deaths were observed, with less than one expected in each of the studies. The results of the other four studies (US pesticide manufacturers [Steenland et al., 1999], Seveso residents [Consonni et al., 2008], and two of the New Zealand pesticide manufacturers ['t Mannetje et al., 2005; McBride et al., 2009]) are summarized in Table 7. Statistically significant excess mortality from multiple myeloma was observed among employees in the first, but not the second of the updated New Zealand pesticide manufacturers cohort, as well as among residents in Seveso zone A. The reduced number of multiple myeloma cases (from 3 to 2) in the New Zealand group suggests that one of the cases originally classified as a multiple myeloma was subsequently reclassified, although this is not clear from the paper. Although results suggest an increased risk of multiple myeloma among people exposed to TCDD (at least for the cohorts of US pesticide manufacturers and Seveso zone A residents, the results have become stronger in the most recent publications), cautious interpretation is warranted due to the lack of detailed results from studies with no cases observed, and the possibility of selective reporting of positive results, all of which are based on small numbers of cases. A recent review of the epidemiology of multiple myeloma reached similar conclusions (Alexander et al., 2007).

**Table 7:** Relative risk of multiple myeloma mortality in selected studies of populations exposed to dioxin—Results reported before and after the 1997 IARC Monograph (IARC, 1997)

	Publication before the 1997 IARC Monograph				Publication after the 1997 IARC Monograph			
		
Population	Reference	O	SMR	95% CI	Reference	O	SMR	95% CI
US pesticide mft	Fingerhut et al., 1991	5	1.6	0.5, 3.9	Steenland et al., 1999	10	2.07	0.99, 3.80
NZ pesticide mft	Kogevinas et al., 1992	1	6.25	0.16, 34.8	't Mannetje et al., 2005	3	5.51	1.14, 16.1
					McBride et al., 2009**	2	2.2	0.2, 8.1
Seveso, Zone A	Bertazzi et al., 1996*0	0	0, *18.4*	Consonni et al., 2008***	2	4.34	1.07, 17.5	
Seveso, Zone B	Bertazzi et al., 1996*	5	*3.3*	*1.1, 7.7*	Consonni et al., 2008***	5	1.68	0.69, 4.10

*Note*. O = observed deaths; mft = manufacturers; SMR = standardized mortality ratio; CI = confidence interval.

* Similar results in the cancer incidence analysis (Bertazzi et al., 1993).

** Unclear why the expanded and updated cohort (McBride et al., 2009) has one less case of the earlier cohort ('t Mannetje et al., 2005).

*** Similar results in the cancer incidence analysis (Pesatori et al., 2009).

Figures in italics were derived from raw data reported in the paper.

### TCDD exposure and risk of breast cancer

The risk of breast cancer from exposure to TCDD has been reviewed in detail by several authors (Laden and Hunter, 1998; Calle et al., 2002; Brody et al., 2007) as well as IARC (IARC, 1997), with no consistent evidence of an increased risk. In particular, no excess breast cancer incidence or mortality was observed in the population-based studies of Seveso residents (Table 8) (Bertazzi et al., 1993, 1996; Consonni et al., 2008). An independent study was conducted in a cohort of 981 women living in zones A and B, with serum samples collected during 1976-1981 (Warner et al., 2002). During 1996-1998 these women were asked whether they had been diagnosed with cancer, and self-reported cases were validated against pathology and medical records. The median serum TCDD of 15 cases of breast cancer (71.8 ppt) was higher than that of the study population (55.1 ppt). The hazard ratio for 10-fold increase in serum TCDD level was 2.1 (95% CI 1.0, 4.6). Limitations of this study include back-extrapolation of serum TCDD level for many participants, and the lack of multivariate adjustment for potential confounders.

**Table 8:** Risk of female breast cancer among residents in Seveso

	Incidence*			Mortality**		
		
	O	SIR	95% CI	O	SMR	95% CI
Zone A	8	1.43	0.71, 2.87	2	0.60	0.15, 2.41
Zone B	30	0.85	0.59, 1.22	13	0.65	0.37, 1.12
Zone R	249	1.00	0.88, 1.15	133	0.87	0.73, 1.05

*Note*. O = observed cases/deaths; SIR, standardized incidence ratio; SMR = standardized mortality ratio; CI = confidence interval.

* Follow-up 1977–1996 (Pesatori et al., 2009).

** Follow-up 1976–2001 (Consonni et al., 2008).

A study of 30,454 wives of farmers from Iowa and North Carolina enrolled in 1993-1997 in the Agriculture Health Study (AHS) and followed up to 2000 identified 309 cases of breast cancer (Engel et al., 2005). Detailed information on the use of 50 pesticides by the women and their husbands was obtained at enrollment via questionnaire. Less than 1% of cases and 0.7% of non-cases ever used 2,4,5-T, the herbicide most likely contaminated by TCDD (no risk estimate available). The RR for use of 2,4,5-T by husbands of women who did not use pesticides was 1.3 (95% CI 0.9, 1.9).

Because occupational cohorts of TCDD-exposed workers included few women, results on breast cancer were limited by small numbers. Community-based case-control studies of breast cancer were limited by imprecise exposure assessment and low prevalence of exposure to dioxin above background (Adami et al., 1995; Calle et al., 2002). Two studies comparing TCDD level in breast adipose tissue among women with cancer and benign disease reported no statistically significant difference (Hardell et al., 1996; Reynolds et al., 2005). In a recent study of 104 male breast cancers and 1901 community controls from eight European countries, the OR for estimated occupational exposure to PCB and dioxin below the median was 0.9 (95% CI 0.3, 2.6), that for exposure above the median was 1.6 (95% CI 0.7,3.7) (Villeneuve et al., 2010).

Overall, the evidence linking TCDD exposure to breast cancer risk is inconclusive at present: a conclusion that is consistent with previous reviews (Adami et al., 1995; Calle et al., 2002).

### Studies of chloracne patients

Groups of individuals who developed chloracne following high TCDD exposure have been studied in the cohorts of US (A/=608), German (AT=113), and Dutch herbicide manufacturers (*N* = 29), as well as Seveso residents (*N* = 182). Among US chloracne patients, 73 cancer deaths occurred (SMR 1.25; 95% CI 0.98, 1.57), including 30 from lung cancer (SMR 1.45; 95% CI 0.98, 2.07), 6 from lymphohematopoietic neoplasms (SMR 1.13; 95% CI 0.41, 2.46), and 3 from STS (SMR 11.32; 95% CI 2.33, 33.10) (Steenland et al., 1999). Among 113 German chloracne patients, 18 cancer deaths were observed 20 or more years since first exposure (SMR 1.90; 95% CI 1.13, 3.00), including 6 from digestive cancers (SMR 1.83; 95% CI 0.67, 3.98) and 7 from respiratory cancers (SMR 2.42; 95% CI 0.97, 4.99) (Ott and Zober, 1996). Cancer risk was higher among patients with moderate chloracne than among those with severe disease. No cancer deaths or cases were identified among Seveso chloracne patients (Consonni et al., 2008; Pesatori et al., 2009). The number of expected cases or deaths was not provided, but it is likely to be lowbecause of the young age of these patients (Baccarelli et al., 2005). No cancer deaths were reported among Dutch chloracne patients (Hooiveld et al., 1998).

## Discussion

We compared the current evidence of cancer risk among individuals exposed to TCDD with the results available at the time of the 1997 IARC Monograph. Since 1997, the strongest evidence for a carcinogenic effect comes from the exposure-response reanalysis of all-cancer mortality among herbicide manufacturing cohort, in which an association was apparent at high doses, or when lagging of exposure was applied (Steenland et al., 1999, 2001). Supportive evidence comes also from the updated mortality follow-up of the Seveso population (Consonni et al., 2008). Nevertheless, other results published since 1997—including the main SMR analysis of the US herbicide manufacturing cohort (Steenland et al., 1999), the updated mortality analysis of one of the largest plants included in the US cohort (Collins et al., 2008a, 2008b), the cancer incidence follow-up of the Seveso population (Pesatori et al., 2009), the update of the Dutch cohort of herbicide manufacturers (Boers et al., 2010), and the studies of Vietnam veterans (Akhtar et al., 2004; Cypel and Kang, 2010)—do not support an association between TCDD exposure and cancer risk.

Among the four studies with highest TCDD exposure, given the greatest weight in the 1997 IARC review (IARC, 1997), updated results were reported for the US multicenter cohort (Steenland et al., 1999, 2001) and the Dutch cohort (Boers et al., 2010). Whereas the US cohort added evidence in favor of an association between TCDD exposure and cancer risk, the Dutch cohort added evidence against it. Although the hypothesis that TCDD is a human carcinogen is plausible based on experimental evidence, in our opinion the weak and contradictory evidence from epidemiologic studies does not support a causal association.

With respect to results for individual neoplasms, the updated mortality analysis of the Seveso population suggested an increased risk of lung cancer (Consonni et al., 2008), which was not confirmed in the update of any other study. As for NHL, the updates of the mortality analyses of the Seveso population (Consonni et al., 2008) and New Zealand herbicide sprayers suggested an increased risk, which was not confirmed in the updates of the US multicenter study (Steenland et al., 1999), the Dutch study (Boers et al., 2010), or the Ranch Hand study (Akhtar et al., 2004). An increased mortality from STS was suggested in the updated mortality analysis of the Midland cohort (Collins et al., 2008a), but not in the analysis of the larger US multicenter cohort (Steenland et al., 1999) or in the other studies.

Because human and experimental studies on TCDD and cancer risk have continued for more than 40 years, this area of research is heavily charged with political and emotional issues. In such circumstances, specific types of bias might occur, in addition to those affecting epidemiologic studies in general. These include publication, diagnostic and reporting biases. Selective reporting of “positive” results is plausible because of the great concern that TCDD is an environmental carcinogen. As has been shown (Boffetta et al., 2008), early studies on NHL risk might have been subject to publication bias. To assess publication bias by identifying unpublished studies is difficult. In the case of TCDD and cancer, there is at least one such example: a case-control study of STS and NHL conducted in the 1990s in Ho Chi Min City, Vietnam, on the possible association with Agent Orange exposure during the Vietnam War (Kramarova et al., 1998). To the best of our knowledge no results have been reported. Furthermore, apart from the findings on breast cancer mentioned above (Engel et al., 2005), and a brief mention of null results in a study of prostate cancer (Alavanja et al., 2003), to our knowledge no results on TCDD-contaminated pesticides have been reported from the AHS, the most extensive study of agricultural exposures and cancer conducted to date.

Bias might also arise because several of the cohorts have been subject to intensive medical surveillance, possibly resulting in overdiagnosis as compared to the unexposed populations used for comparison. This potential form of bias would be particularly relevant for neoplasms whose detection is highly dependent on diagnostic intensity (e.g., melanoma, and thyroid and prostate cancer [Adami, 2010]), and for neoplasms suspected to be linked to dioxin exposure, which are subject to diagnostic uncertainties, such as STS and NHL, as has been suggested for pleural mesothelioma in cohorts exposed to asbestos (Siemiatycki and Boffetta, 1998). However, no direct evidence is available in favor of, or against, this form of bias.

Two of the most informative studies (US herbicide manufacturers and Seveso residents) suggest an association with all cancer only after a latency of 15 or 20 years (Steenland et al., 1999, 2001; Consonni et al., 2008; Pesatori et al., 2009). The authors interpret these studies as supporting a causal association between TCDD and cancer risk. TCDD exerts its carcinogenic effect in experimental systems through the Ah receptor, which activates cell proliferation (Safe, 2001). This mechanism has been invoked to explain an unspecific action on risk of all cancer (Mandal, 2005; Walker, 2007). However, a long latency between exposure and cancer is a hallmark of agents acting through DNA damage. Cheng and colleagues (2006) have proposed to solve the apparent inconsistency by assuming that cell proliferating activity of TCDD should be sustained for a long follow-up. Hence, individuals with less than 10 or 15 years since first exposure would not have acquired a sufficiently long exposure. However, known carcinogens acting via other late-stage mechanisms (e.g., hormones) do not support this notion.

A more fundamental challenge in the evaluation of TCDD as a human carcinogen is the emphasis on risk of all cancer rather than specific neoplasms or a specific subset of neoplasms. As already explained in a previous review (Cole et al., 2003), we have not found convincing evidence for a central role of the Ah receptor in dioxin-related carcinogenesis. Hence, the ubiquitous presence of the Ah receptor should not guide the interpretation of the epidemiologic evidence we have summarized above. Indeed, a non-organ-specific carcinogenicity of TCDD would represent a unique feature in cancer epidemiology. Known nongenotoxic carcinogenic agents, including those acting through pathways present in all organs and tissues (e.g., overweight/obesity [World Cancer Research Fund, 2007]), target only one or a few specific organs.

An increase in all-cancer risk has not been clearly shown in animal carcinogenicity tests. The reader is referred to previous reviews (IARC, 1997; US EPA, 2003; Knerr, 2006) and results of recent studies (NTP, 2006). TCDD causes specific tumors in mice (hepatocellular carcinoma, skin tumors, and lymphoma in multiple studies, as well as thyroid and lung tumors in single studies), rats (hepatocellular carcinoma, cholangiocarcinoma, and lung and oral cavity tumors in multiple studies, as well as tumors of the thyroid, skin, and uterus in single studies), and hamster (skin tumors in multiple studies). TCDD is therefore classified as an experimental carcinogen (IARC, 1997; US EPA, 2003; Baan et al., 2009); however, these evaluations are based on the evidence of increased incidence of groups of specific tumors (and in particular those commonly occurring in mice and rats), and not on all-cancer incidence in experimental animals.

Consistency of results is a key criterion for assessing causality. Shifting the emphasis from specific cancers to all cancer allows for the possibility that different types of cancer-specific biases affecting various studies (e.g., residual confounding by different risk factors) would generate an apparently consistent increase in all-cancer risk. Protection from bias should therefore be subject to more stringent scrutiny when evaluating an epidemiological hypothesis that some risk factor causes all cancers.

In addition, the hypothesis of an increase in all-cancer risk would not dispense with the requirement that cancer-specific results have to be consistent across studies, especially in the case of more common cancers, such as lung cancer, for which random fluctuation can hardly be invoked as cause for the lack of consistency. For none of the specific neoplasms is there a consistent pattern showing an increased risk in populations exposed to TCDD.

In some of the epidemiologic studies, an exposure-response is suggested, in the absence of an overall increase in cancer risk. In these circumstances, the apparent trend arises from a comparison of exposure categories with SMRs distributed below and above the null value of 1.0. One example is lung cancer mortality in the cohort of US chemical workers: the overall SMR was 1.06 (95% CI 0.88,1.26), and the *p* value of test for trend in an internal analysis by cumulative exposure score (log-transformed and lagged 15 years) was .03 (Steenland et al., 1999). In this analysis, the SMR of three of the seven exposure categories was below 1.0.

A further problem in the interpretation of the epidemiologic studies of TCDD exposure and cancer risk arises from the fact that mortality was used as the outcome measure in several of them. In addition to possible differential misclassification of causes of death between exposed and unexposed subjects, there are other issues, including confounding by different quality of treatment among exposed and unexposed due to different access to health care. Another problem in population-based studies such as the cohort of Seveso residents is that mortality analysis is not incidence based. Hence, there is a contribution to the mortality from cases that were already prevalent at the time of exposure. Conversely, when occupational cohorts are compared with the general population, a healthy worker effect is quite likely due to a lower prevalence of existing cancer among those who are occupationally active.

In conclusion, the carcinogenicity of TCDD may be plausible on the basis of animal experiments conducted at high doses, but the epidemiological evidence falls far short from conclusively demonstrating such a relationship in humans. In the case of complex data such as the epidemiologic studies on TCDD exposure and cancer risk, it is important to consider all the evidence, and not just selected components that might support one particular hypothesis. The use of mechanistic data to link experimental and human results is justified when there is a specific and strong correspondence between the different systems such as presence of the same DNA damages and mutations in the same cancer-related genes in both human tumors, experimental animals and in vitro systems, as in the case of benzo[a]pyrene, alterations in *TP53* gene, and lung cancer (IARC, 2010). The exercise becomes questionable, however, when weaker data are used and inconsistencies are ignored. Furthermore, TCDD is a likely example of how epidemiologic studies in a controversial area might be particularly susceptible to multiple types of bias. These considerations support our conclusion that the epidemiological evidence of carcinogenicity of TCDD in humans is not “sufficient” and remains “limited.”

## Footnotes

1 Additional papers published shortly after the 1997 IARC Monograph on the 4-plant German cohort (Becher et al., 1998; Flesch-Janys et al., 1998), and the Danish cohort (Lynge et al., 1998) were based on the same data included in the multicenter study (Kogevinas et al., 1997), and therefore are not reviewed in detail here.

2 An additional report of mortality among the workers in this factory has been published (Bodner et al., 2003).

3 Additional reports based on follow-up of cancer incidence to 1991 and mortality to 1996 (Bertazzi et al., 2001; Pesatori et al., 2003) are not reviewed in detail.

4 An additional report based on cancer mortality to 1993 (Michalek et al., 1998) is not reviewed in detail.

5 These results were derived by summing up observed and expected deaths in White and non-White veterans reported in the original publication.
